# Dataset on modeling and optimization analysis of biodegradation of paracetamol

**DOI:** 10.1016/j.dib.2020.105826

**Published:** 2020-06-07

**Authors:** Sunil Chopra, Dharmender Kumar

**Affiliations:** Department of Biotechnology, Deenbandhu Chhotu Ram University of Science and Technology, Murthal-131039 Sonepat, Haryana, India

**Keywords:** Acetaminophen, Biodegradation, Box-behnken design (BBD), Design expert^Ⓡ^ software, Wastewater

## Abstract

This article contains the experimental and statistical data related to degradation of acetaminophen (paracetamol, APAP) by bacterial strains. The strains used in this study were isolated from wastewater by enrichment culture method. The optimization was important to identify the physical conditions at which the strain degraded the APAP effectively. Therefore, the Box-Behnken design (BBD) was used to know the influence of physical parameters (*viz*. pH, temperature, agitation speed, and concentration) on the degradation of APAP. The effects of the physical factor on the degradation process were investigated by a mathematical model, and this had indicated that all physical factors having some effect on the biodegradation of the APAP. Analysis of variance (ANOVA) showed that the strains DPP1, DPP3, DKP1, and DKP2 had the F-value of 12.89, 6.45, 4.58, and 5.31, respectively. This indicated, the model was significant with regression coefficient (R) value of 0.01%, 0.06%, 0.37%, and 0.18%, respectively. The experimental values, predicted data, and ANOVA analysis has suggested that the model was satisfactory.

**Specifications table**

**Table:** 

Subject area	Environmental science
More specific subject area	Biodegradation
Type of data	Table and Figures
How data was acquired	The bacterial strains*viz.Staphylococcus sciuri* DPP1 (MN744326), *Bacillus subtilis* DPP3 (MN744327), *Bacillus paralicheniformis* DKP1 (MN744324) and *Enterococcus faecium* DKP2 (MN744325) were isolated from sewage water, has the potential to degrade APAP in shake flask. Further, to know the effect of physical factors (*viz*. pH, temperature, agitation speed, and concentration of APAP) on degradation Box-Behnken design was used for the optimization of experimental conditions.
Data format	Raw (Table 1) and analyzed (Table 2)
Parameters for data collection	Physical factors used for degradation of APAP were, pH (3- 11), temperature (10- 70 °C), agitation speed (50- 250 rpm), and concentration of APAP(20- 1200 mg/L). Statistical analysis of biodegradation of APAP, using Box-Behnken design (BBD). The 3D- plots indicated the effect of physical factors on biodegradation.
Data source location	DeenbandhuChhotu Ram University of Science and Technology, Murthal-131,039, Sonepat, Haryana, India.
Data accessibility	Data information is available in this article only.
Related research articles	Chopra S, Kumar D (2020) Characterization, optimization and kinetics study of acetaminophen degradation by Bacillus drentensis strain S1 and waste water degradation analysis. Bioresour Bioprocess 7:. https://doi.org/10.1186/s40643–020–0297-x

## Value of the data

This data analysis was focused on the optimization of physical parameters *viz*. pH, temperature, agitation speed, and concentration of APAP, employed for the degradation of APAP. The degradation efficiency of strains can be increased by performing degradation at optimal physical conditions. This is not only eco-friendly but also a cost-effective technique for the removal of such compounds with better efficiency.
The statistical data will be useful for the optimization of the degradation of APAP from wastewater.
The data will be further used for improving the degradation of APAP using co-degradation, and the effect of various nutrients on the degradation of strains at the optimal physical conditions.
BBD of Design Expert^Ⓡ^ software was successfully used to design the experiments. After that, the predicted value for the biodegradation of APAP, was predicted by it. Further, this helps in the optimization of parameters which reduces the number of runs required to perform the experiment.

## Data description

**Fig. 1:**
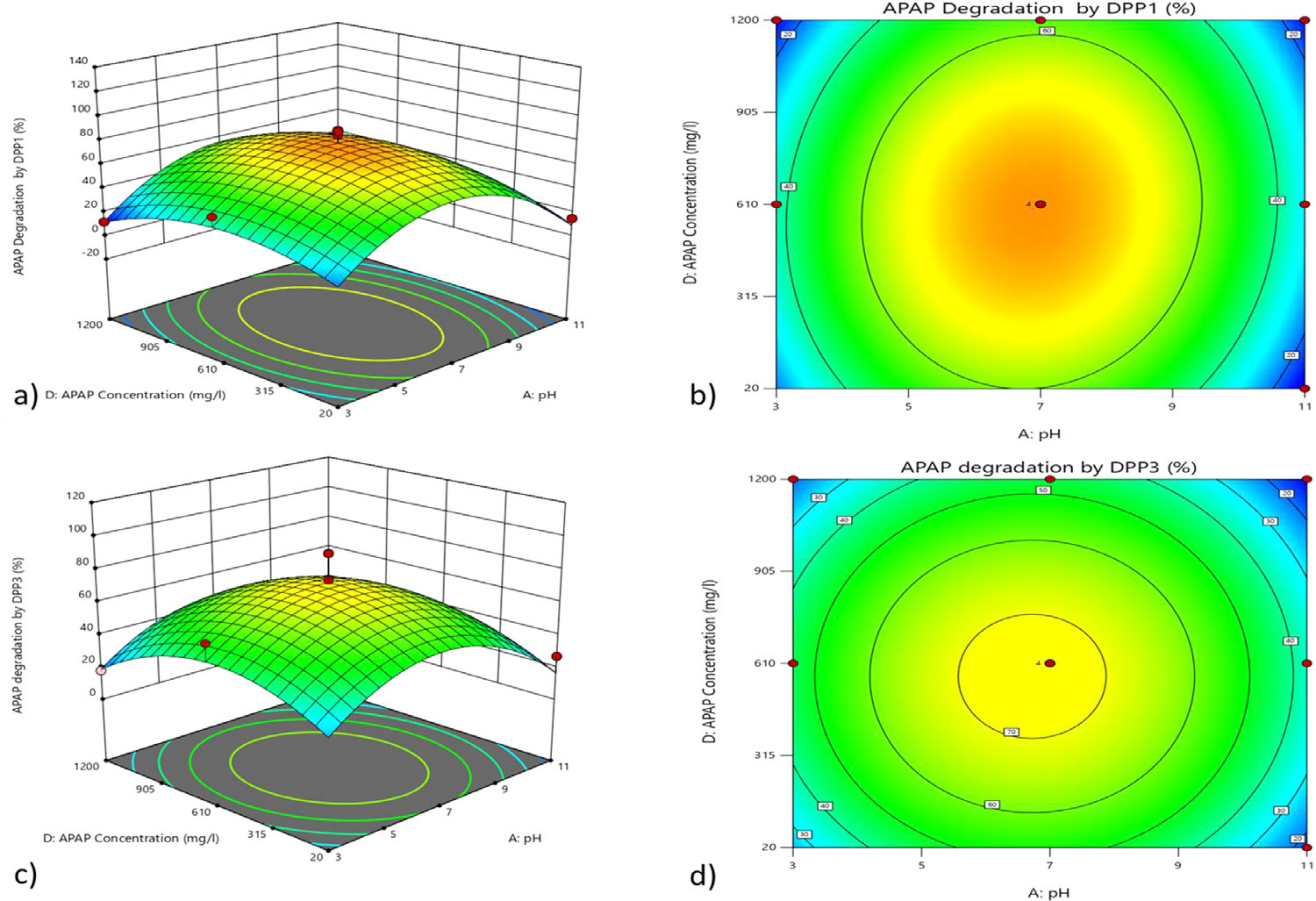
The contour plots and 3D-plots between physical parameter, A: temperature, B: pH, C: Agitation speed and D: concentration of APAP a) The 3D- plots showing APAP degradation by DPP1 between D and A,b)The contour plots showing APAP degradation by DPP1 between D and A, c)The 3D- plots showing APAP degradation by DPP3 between D and A,d) The contour plots showing APAP degradation by DPP3 between D and A.

**Fig. 2:**
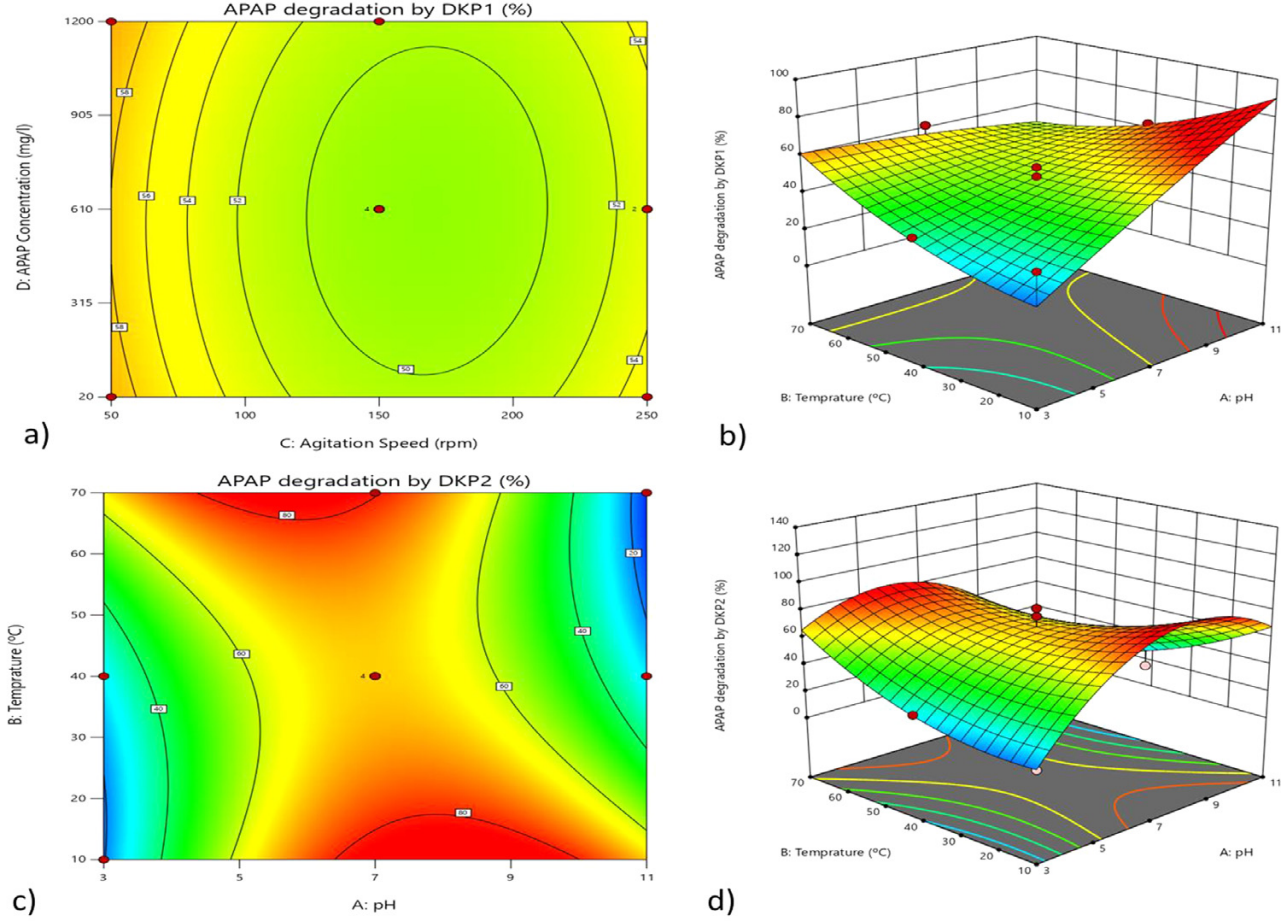
The contour plots and 3D-plots between physical parameter, A: temperature, B: pH,C: agitation speed and D: concentration of APAP a)The contour plots showing APAP degradation by DKP1 between D and C, b)The 3D- plots showing APAP degradation by DKP1 between Band A, c)The contour plots showing APAP degradation by DKP2 between Band A,d) The 3D- plots showing APAP degradation by DKP2 between Band A.

The data represented the use of Box-Benkin Design(BBD) for the optimization of physical condition for the degradation of paracetamol (APAP) also known as acetaminophen by four bacterial strains. These strains were isolated from sewage sources using enrichment culture methods [1]. The physical factors pH, temperature, agitation speed, and concentration of APAP were used to understand the degradation (Table 1). The model suggested 29 experiments with varied physical factors predicted through BBD of Design Expert^Ⓡ^ software (Design-Expert^Ⓡ^ Version 12.0.3.0; State-ease, Inc.) (Table 2). Further, the analysis of variance (ANOVA) was predicted for each strain. This model suggested that the F-value of 12.89 indicated that the model is significant for DPP1 and 0.01% chance in the F-value due to noise ratio. P-values less than 0.0500 indicated that the model was significant and B, A², C², D² were the significant model terms. The lack of fit F-value of 0.41 indicated the lack of fit was not significant relative to pure error (Table 3). Similarly, through ANOVA, it was concluded that for the bacterial isolates DPP3 (Table 4), DKP1 (Table 5), and DKP2 (Table 6).The F-valueof 6.45, 4.58, and 5.31, respectively. The significance with DPP3 (0.06%), DKP1(0.37%), and DKP2(0.18%) (Tables 4,5,6,). Further, the contour plots and 3-D plots showing the APAP degradation between physical factors, *viz*. A: temperature, B: pH, C: Agitation speed and D: concentration of APAP were constructed between various parameters like The 3D- plots showing APAP degradation: by DPP1 between D and A, (Fig 1a), by DPP3 between D and A (Fig 1c), by DKP1 between B and A(Fig 2b),by DKP2 between B and A (Fig 2d),etc. Similarly, the contour plots showing APAP degradation by DPP1 between D and A (Fig 1b),by DPP3 between D and A (Fig 1d), by DKP1 between D and C (Fig 2a), by DKP2 between B and A (Fig 2c), etc. The P-values less than 0.0500 for each strain indicates that the model was significant with B, A², C², D² are significant model terms for DPP3; A is a significant model term for DKP1 and A², D² are significant model terms for DKP2. The **l**ack of fit F-value for DPP3, DKP1, and DKP2 of 0.56, 1.48, and 0.64, respectively. This has suggested that the lack of fit was not significant relative to the pure error. Finally, the solution table was generated by the BBD-quadratic model. This table suggested that the strains DPP1, DPP3, DKP1, and DKP2 have the optimal pH at 7.6,4.1, 6.9, and 6.1 respectively, and the optimal temperaturewas at 47 °C, 37 °C, 11 °C, and 53 °C respectively. Similarly, the model suggested optimal agitation speed was at 140 rpm, 115 rpm, 77 rpm and 161 rpm, respectively and the concentration of APAP in mg/L was at 886, 1171, 558, and 1065, respectively.

**Table 1:** Physical factors and experimental ranges for experiments.

Factor code	Factor	Units	Box-Behnken Design			
			Low (−1)	High (+1)	Mean	Std. Dev.
A	pH		3.00	11.00	7.00	2.62
B	T emp	°C	10.00	70.00	40.00	19.64
C	Agitation Speed	rpm	50.00	250.00	150.00	65.47
D	APAP Concentration	mg/l	20.00	1200.00	610.00	386.25

**Table 2:** Experimental design and individual factor study using box-behnken design, and corresponding response for APAP biodegradation.

		Factor 1	Factor 2	Factor 3	Factor 4	Response 1		Response 2		Response 3		Response 4	
Std	Run	A:pH	B:Temp	C:Agitation Speed	D:APAP Concentration	APAP Degradation by DPP1		APAP degradation by DPP3		APAP degradation by DKP1		APAP degradation by DKP2	
				rpm	mg/L	%		%		%		%	
						Observed	Predicted	Observed	Predicted	Observed	Predicted	Observed	Predicted
26	1	7	40	150	610	89	80.60	74	75.00	47	51.00	82	70.80
29	26	7	40	150	610	88	43.04	90	50.67	43	24.96	76	35.54
25	22	7	40	150	610	85	11.17	67	17.13	54	67.58	76	14.33
28	24	7	40	150	610	84	12.17	92	17.29	62	35.25	78	20.00
15	28	7	10	250	610	79	10.04	76	13.33	48	27.79	68	16.54
16	25	7	70	250	610	74	62.79	82	60.71	57	58.13	80	56.96
21	6	7	10	150	20	67	41.17	24	31.13	38	47.75	42	38.50
13	9	7	10	50	610	67	66.79	63	68.04	57	56.13	68	65.63
23	8	7	10	150	1200	65	74.67	62	70.96	58	66.42	72	74.00
27	15	7	40	150	610	57	9.67	52	3.46	49	59.58	42	8.00
8	21	7	40	250	1200	57	41.29	52	36.21	49	49.96	45	45.13
5	27	7	40	50	20	56	16.29	38	10.21	62	39.79	39	19.13
22	18	7	70	150	20	47	13.29	52	8.38	49	63.13	42	10.63
1	2	3	10	150	610	45	13.67	56	9.96	34	26.25	29	7.33
7	29	7	40	50	1200	43	80.60	27	75.00	65	51.00	39	70.80
6	23	7	40	250	20	36	27.54	23	31.33	57	69.29	26	29.04
14	7	7	70	50	610	35	20.54	32	26.00	46	49.13	39	18.04
24	11	7	70	150	1200	34	48.29	26	49.87	45	46.96	36	47.46
2	16	11	10	150	610	21	23.79	24	25.54	63	25.96	15	17.46
20	20	11	40	250	610	19	21.79	16	20.71	69	68.29	14	20.96
4	17	11	70	150	610	18	60.04	14	56.17	42	56.63	13	57.54
18	13	11	40	50	610	17	80.60	15	75.00	67	51.00	18	70.80
19	19	3	40	250	610	17	44.54	15	36.83	26	57.63	11	38.88
3	5	3	70	150	610	16	80.60	14	75.00	36	51.00	19	70.80
17	12	3	40	50	610	16	62.67	11	63.46	43	53.42	27	63.33
10	3	11	40	150	20	15	80.60	27	75.00	69	51.00	28	70.80
12	10	11	40	150	1200	14	53.54	11	45.50	67	60.46	13	50.04
9	14	3	40	150	20	13	69.17	23	66.29	19	52.08	13	57.83
11	4	3	40	150	1200	12	35.04	18	19.83	28	62.46	17	37.71

**Table 3:** Analysis of variance (ANOVA) for the APAP Degradation by DPP1.

Source	Sum of Squares	df	Mean Square	F-value	p-value	
Model	18,729.77	14	1337.84	12.89	< 0.0001	significant
A-pH	18.75	1	18.75	0.1807	0.6772	
B-Temp	1200.00	1	1200.00	11.56	0.0043	
C-Agitation Speed	192.00	1	192.00	1.85	0.1953	
D-APAP Concentration	6.75	1	6.75	0.0650	0.8024	
AB	169.00	1	169.00	1.63	0.2227	
AC	0.2500	1	0.2500	0.0024	0.9615	
AD	0.0000	1	0.0000	0.0000	1.0000	
BC	182.25	1	182.25	1.76	0.2063	
BD	30.25	1	30.25	0.2915	0.5977	
CD	289.00	1	289.00	2.78	0.1174	
A²	15,712.09	1	15,712.09	151.41	< 0.0001	
B²	240.70	1	240.70	2.32	0.1500	
C²	1028.43	1	1028.43	9.91	0.0071	
D²	2521.60	1	2521.60	24.30	0.0002	
Residual	1452.78	14	103.77			
Lack of Fit	739.58	10	73.96	0.4148	0.8819	not significant
Pure Error	713.20	4	178.30			
Cor Total	20,182.55	28

**Table 4:** Analysis of variance (ANOVA) for theAPAP degradation by DPP3.

Source	Sum of Squares	df	Mean Square	F-value	p-value	
Model	17,180.20	14	1227.16	6.45	0.0006	significant
A-pH	33.33	1	33.33	0.1752	0.6819	
B-Temp	1365.33	1	1365.33	7.18	0.0180	
C-Agitation Speed	574.08	1	574.08	3.02	0.1043	
D-APAP Concentration	30.08	1	30.08	0.1581	0.6969	
AB	256.00	1	256.00	1.35	0.2654	
AC	2.25	1	2.25	0.0118	0.9149	
AD	110.25	1	110.25	0.5796	0.4591	
BC	342.25	1	342.25	1.80	0.2012	
BD	110.25	1	110.25	0.5796	0.4591	
CD	506.25	1	506.25	2.66	0.1251	
A²	12,110.01	1	12,110.01	63.66	< 0.0001	
B²	13.80	1	13.80	0.0725	0.7916	
C²	1575.18	1	1575.18	8.28	0.0122	
D²	2551.53	1	2551.53	13.41	0.0026	
Residual	2663.25	14	190.23			
Lack of Fit	1555.25	10	155.53	0.5615	0.7908	not significant
Pure Error	1108.00	4	277.00			
Cor Total	19,843.45	28

**Table 5:** Analysis of variance (ANOVA) for theAPAP degradation by DKP1.

Source	Sum of Squares	df	Mean Square	F-value	p-value	
Model	4608.88	14	329.21	4.58	0.0037	significant
A-pH	3234.08	1	3234.08	45.00	< 0.0001	
B-Temp	225.33	1	225.33	3.14	0.0984	
C-Agitation Speed	56.33	1	56.33	0.7839	0.3909	
D-APAP Concentration	0.7500	1	0.7500	0.0104	0.9201	
AB	132.25	1	132.25	1.84	0.1964	
AC	90.25	1	90.25	1.26	0.2813	
AD	72.25	1	72.25	1.01	0.3330	
BC	100.00	1	100.00	1.39	0.2578	
BD	6.25	1	6.25	0.0870	0.7724	
CD	2.25	1	2.25	0.0313	0.8621	
A²	310.32	1	310.32	4.32	0.0566	
B²	10.82	1	10.82	0.1506	0.7038	
C²	175.96	1	175.96	2.45	0.1400	
D²	61.67	1	61.67	0.8581	0.3700	
Residual	1006.08	14	71.86			
Lack of Fit	792.08	10	79.21	1.48	0.3758	not significant
Pure Error	214.00	4	53.50			
Cor Total	5614.97	28

**Table 6:** Analysis of variance (ANOVA) for theAPAP degradation by DKP2.

Source	Sum of Squares	df	Mean Square	F-value	p-value	
Model	14,630.02	14	1045.00	5.31	0.0018	Significant
A-pH	18.75	1	18.75	0.0953	0.7620	
B-Temp	675.00	1	675.00	3.43	0.0851	
C-Agitation Speed	56.33	1	56.33	0.2865	0.6009	
D-APAP Concentration	30.08	1	30.08	0.1530	0.7016	
AB	16.00	1	16.00	0.0814	0.7796	
AC	36.00	1	36.00	0.1831	0.6753	
AD	90.25	1	90.25	0.4589	0.5092	
BC	420.25	1	420.25	2.14	0.1659	
BD	30.25	1	30.25	0.1538	0.7008	
CD	240.25	1	240.25	1.22	0.2877	
A²	12,382.45	1	12,382.45	62.96	< 0.0001	
B²	34.81	1	34.81	0.1770	0.6803	
C²	657.33	1	657.33	3.34	0.0889	
D²	1400.08	1	1400.08	7.12	0.0184	
Residual	2753.22	14	196.66			
Lack of Fit	1692.42	10	169.24	0.6382	0.7432	not significant
Pure Error	1060.80	4	265.20			
Cor Total	17,383.24	28

## Experimental design, materials, and methods

### Materials

The acetaminophen (99% pure) was obtained from Sigma Aldrich (USA) and all other highly pure chemicals were purchased from HiMedia (Mumbai, India), to perform degrading experiments. The strains used in this data analysis were isolated from the wastewater flow in the drains present in Sonipat, Panipat, Karnal, and Yamunanagar (Haryana, India); Delhi, India [1].

### Design of experiment

Primarily, the experiments were designed with Box-Behnken design (BBD) Design expert^Ⓡ^ (Design-Expert^Ⓡ^ Version 12.0.3.0; State-ease, Inc.). In the model, four variables (physical factors) were used and a total of 29 experiments were designed [2]. The four physical factors used were pH (A), temperature (B), agitation speed (C), and concentration of APAP (D). Further, the response variables, APAP degradation by DPP1, DPP3, DKP1, and DKP2, were determined through experiments conducted in the lab and by system responses. After that, the mathematical model, ANOVA was applied, and finally, the creation of response surface method plots.The main goal to optimize the maximum degradation under physical factors was evaluated through the interactions between these factors, and modeling mathematical data.

The degradation of APAP was monitored with a UV spectrophotometer at OD_254_ using the colorimetric method [1,3,4]. The degradation percentage (R) of APAP was calculated by Eq. (1):

(1) $$R=\frac{C_{0}-C_{t}}{C_{0}}\times \;100$$

Here, *C*_0_ is the absorbance at the initial concentration of APAP and *Ct,* is the absorbance after incubation at time.

## Declaration of Competing Interest

The authors declare that they have no known competing financial interests or personal relationships that could have appeared to influence the work reported in this paper.

## Appendix Supplementary materials

Image, application 1
